# Comprehensive analysis of geographic and breed-purpose influences on genetic diversity and inherited disease risk in the Doberman dog breed

**DOI:** 10.1186/s40575-023-00130-3

**Published:** 2023-06-05

**Authors:** Claire M. Wade, Robin Nuttall, Sophie Liu

**Affiliations:** 1 School of Life and Environmental Sciences, The University of Sydney, Camperdown, NSW 2006 Australia; 2 Barn Hunt Association LLC, Murray, KY 42071 USA; 3 Doberman Diversity Project, Oakland, CA USA

**Keywords:** Doberman, Diversity, Disorder, Population genetics

## Abstract

**Background:**

Publicly available phenotype data and genotyping array data from two citizen science projects: “Doberman Health Surveys” and “The Doberman Diversity Project” were analyzed to explore relative homozygosity, diversity, and disorder risk according to geographical locale and breeding purpose in the Doberman.

**Results:**

From the phenotypic data cohort, life expectancy of a Doberman at birth is 9.1 years. The leading causes of death were heart disease (accounting for 28% of deaths) and cancers (collectively accounting for 14% of deaths).

By genotyping, the world Doberman population exists as four major cohorts (European exhibition-bred, Americas exhibition-bred, European work, Americas pet/informal). Considering the entire Doberman population, four genomic regions longer than 500 Kb are fixed in 90% or more of 3,226 dogs included in this study. The four fixed regions reside on two autosomal chromosomes: CFA3:0.8–2.3 Mb (1.55 Mb); CFA3: 57.9–59.8 Mb (1.8 Mb); CFA31:0–1.2 Mb (1.2 Mb); and CFA31:4.80–6.47 Mb (1.67 Mb). Using public variant call files including variants for eight Doberman pinschers, we observed 30 potentially functional alternate variants that were evolutionarily diverged relative to the wider sequenced dog population within the four strongly homozygous chromosomal regions.

Effective population size (*Ne*) is a statistical measure of breed diversity at the time of sampling that approximates the number of unique individuals. The major identified sub-populations of Dobermans demonstrated *Ne* in the range 70–236. The mean level of inbreeding in the Doberman breed is 40% as calculated by the number of array variants in runs of homozygosity divided by the assayed genome size (excluding the X chromosome). The lowest observed level of inbreeding in the Dobermans assayed was 15% in animals that were first generation mixes of European and USA bred Dobermans. Array variant analysis shows that inter-crossing between European and USA-bred Dobermans has capacity to re-introduce variation at many loci that are strongly homozygous.

**Conclusions:**

We conclude that efforts to improve breed diversity first should focus on regions with the highest fixation levels, but managers must ensure that mutation loads are not worsened by increasing the frequencies of rarer haplotypes in the identified regions. The analysis of global data identified regions of strong fixation that might impact known disorder risks in the breed. Plausible gene candidates for future analysis of the genetic basis of cardiac disease and cancer were identified in the analysis.

**Supplementary Information:**

The online version contains supplementary material available at 10.1186/s40575-023-00130-3.

## Background

The Doberman dog breed was founded in the late nineteenth century by German individual Karl Friedrich Louis Dobermann and was initially developed as a dog for personal protection in Mr Dobermann’s work as a tax collector. The breed foundation is said to include Pinscher, Weimaraner, Rottweiler and German Shepherd dog breeds and was recognised by the German Kennel Club in 1899 (The Kennel Club https://www.thekennelclub.org.uk/search/breeds-a-to-z/breeds/working/dobermann/ accessed September 7 2021). Since its founding, the intelligence and trainability of the breed has been valued by military and police forces. The breed was first exported from Germany in the 1920s and since then, the breed has had a purpose divided between the original breed working function and as a family companion. As is common in working dog breeds, division of purpose has resulted in differences in breeding objectives between breeders producing animals fit for each purpose. To enhance the protective demeanour of the animal, conformation showing rewards an upright head and neck carriage (specifically referred to as having a general appearance of “proud carriage”), and breeders in some countries have employed tail and or ear docking. Docking is used on the breed until the current time in the USA and Canada but has been eschewed by Federation Cynologique Internationale (FCI) and The Kennel Club (KC) standards since 2015. Differences in breed standards between countries have the potential to affect genes underlying traits in the standards, including conformation of the head carriage, ears and tail in dogs bred for exhibition and temperament for working dogs. Over time the name of the breed has been simplified from Dobermann pinscher to Doberman.

The breed standards and breeding practices of many pedigreed dog breeds have been closely scrutinized in recent times. This scrutiny has revealed potential negative impacts of cosmetic procedures on dog health and welfare (for example, through tail-docking or ear-cropping, or alternatively breeding for tail-less) and negative impacts of breeding practices relating to desirable phenotypes on dog health and longevity [1]. Breeding for novel phenotypes excluded from the breed standard (for example rare coat color or variation in size e.g., giant or miniature) is more common in animals bred for purposes other than exhibition.

Genome-wide homozygosity has been negatively associated with breed mean lifespan (after correction for body-size) and positively associated with disease risk [2–6]. In a study of Kennel Club registered dog breeds in the UK, of 25 breeds with age at death registered for more than 50 individuals, the Doberman had the shortest observed lifespan [7]. Breed mean statistics do not consider geographic, or purpose-bred cohort differences in fitness traits. For example, it is commonly presumed that animals bred for exhibition suffer more negative health consequences due to cosmetic breeding practices and inbreeding than other breed-purpose groups. However, analyses that test these common presumptions are currently lacking.

Breed enthusiasts come from all walks of life, and many are highly educated in scientific findings that are relevant to the health of their breeds. Public declarations may be made regarding the health and genetics of particular breeds with little or no hard data. When data are available, they may be scant or reflect the influences of locale of the reporting researchers or be affected by the individual animals to which researchers have access for DNA collection or phenotypic information. Citizen science projects initiated by breed enthusiasts seek to address deficiencies in relevant information pertaining to the breed of interest by gaining the participation of a large number of interested study participants from many regions of the world.

The current analysis thoroughly investigates the locale and breed-purpose population genomics (disease-associated variant allele frequency, genomic heterozygosity, and genomic sweep) and co-occurrence with disease risk across the Doberman pinscher dog breed. Information generated in this analysis can be used to design measures to improve breed genetic health in the future and may provide some information to identify disorder risk loci including those that might differentially affect individual breed sub-groups.

## Results

### Locale-purpose cohort-based phenotype data

**Fig. 1:**
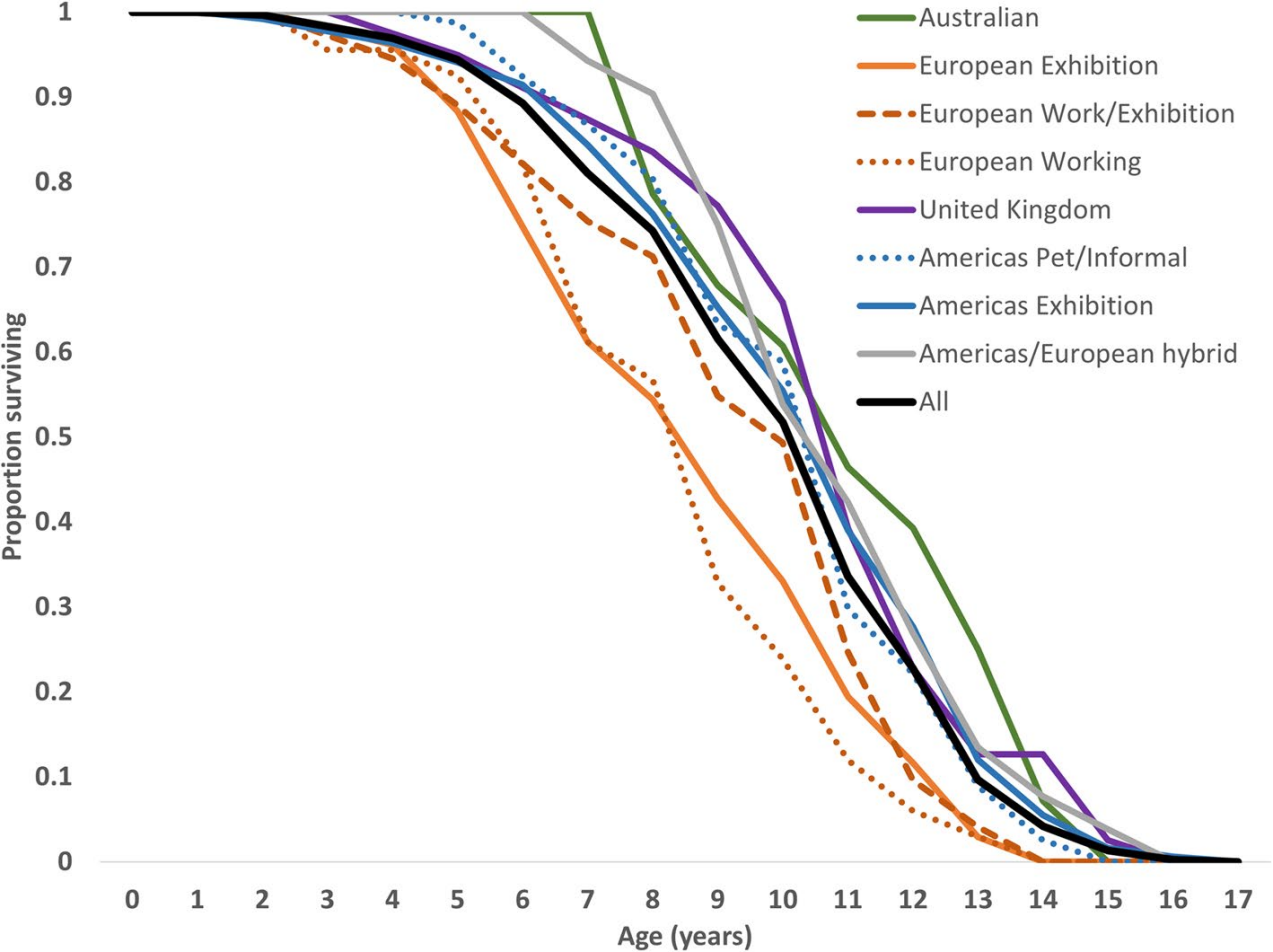
Survival curves for locale-purpose cohorts phenotyped

A life table (Table S1) was constructed using 1,738 reported ages at death. The table reveals that at birth, the life expectancy for a Doberman is approximately 9.1 years. The oldest reported age at death was 17.25 years. In general, dogs born in Europe have shorter life expectancy than those born elsewhere (Fig. 1). At least some of this reduction in life expectancy appears to be attributable to inbreeding depression, since dogs that are of mixed European exhibition and working lines exhibit extended lifespans over the contributing populations.

**Fig. 2:**
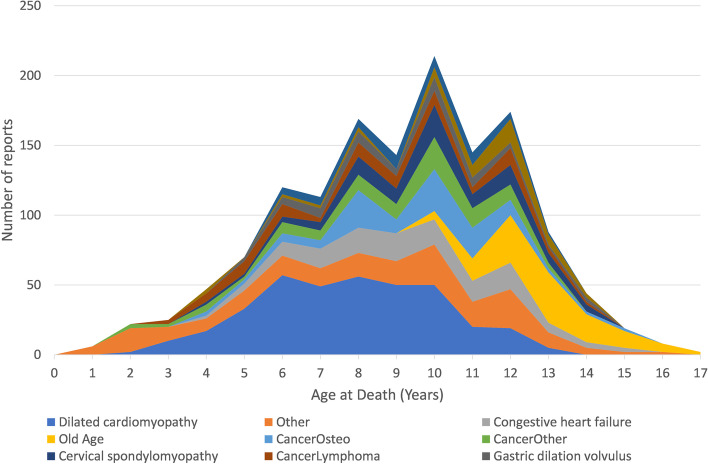
Leading causes of death by age in the Doberman dog breed (causes contributing more than 50 deaths from 1,653 reports)

After data cleaning and restricting analysis to the leading causes of death, 1,653 reported causes of death remained. The leading causes of death according to dog age in years at the time of death across all locales are described in Fig. 2. Major identifiable disorders leading to death in the reported animals are dilated cardiomyopathy, congestive heart failure, osteosarcoma, cervical spondylomyelopathy (wobblers) – here abbreviated to CS, lymphoma, gastric dilation volvulus (bloat), spondylosis and haemangiosarcoma. Percentages of dogs in each locale-purpose group succumbing to leading causes of death are listed in Table 1. Locale-purpose groups with statistically different frequencies of leading causes of death are highlighted for individual cohorts that were identifiable in our genotyping data.

**Table 1:**
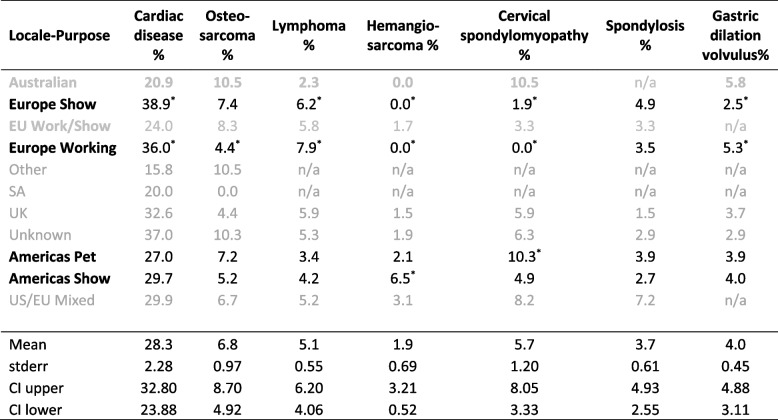
Percentage of deaths attributable to leading causes normalised for regional representation. Grayed text locale-purpose cohorts are not explicitly identifiable in genotyped sample

^*^Percentages outside of the confidence interval (significant deviation from pan-population mean) in locale-purpose groups that are identifiable in the genotyping data

The phenotypic survey data reports that the American and European populations have differential risk of cardiac disease and gastric dilatation volvulus (Table 1). In general, the European exhibition and working populations appeared to have similar disorder risk. No consistent pattern of disorder risk was observed to differentiate the exhibition purpose from other purpose types, although the Americas pet sub-population was reported as having a significantly increased risk of cervical spondylopmyelopathy (CS) and the European working population had significantly lower risk of osteosarcoma than other sub-groups.

### Genotyping array data

The DNA based analysis using Illumina canine genotyping arrays included 216,184 variants and 3,226 dogs (1,489 males, 1,737 females). The total genotyping rate was 0.986 and all dogs passed quality filtering.

### Locale-purpose based DNA profiles

**Fig. 3:**
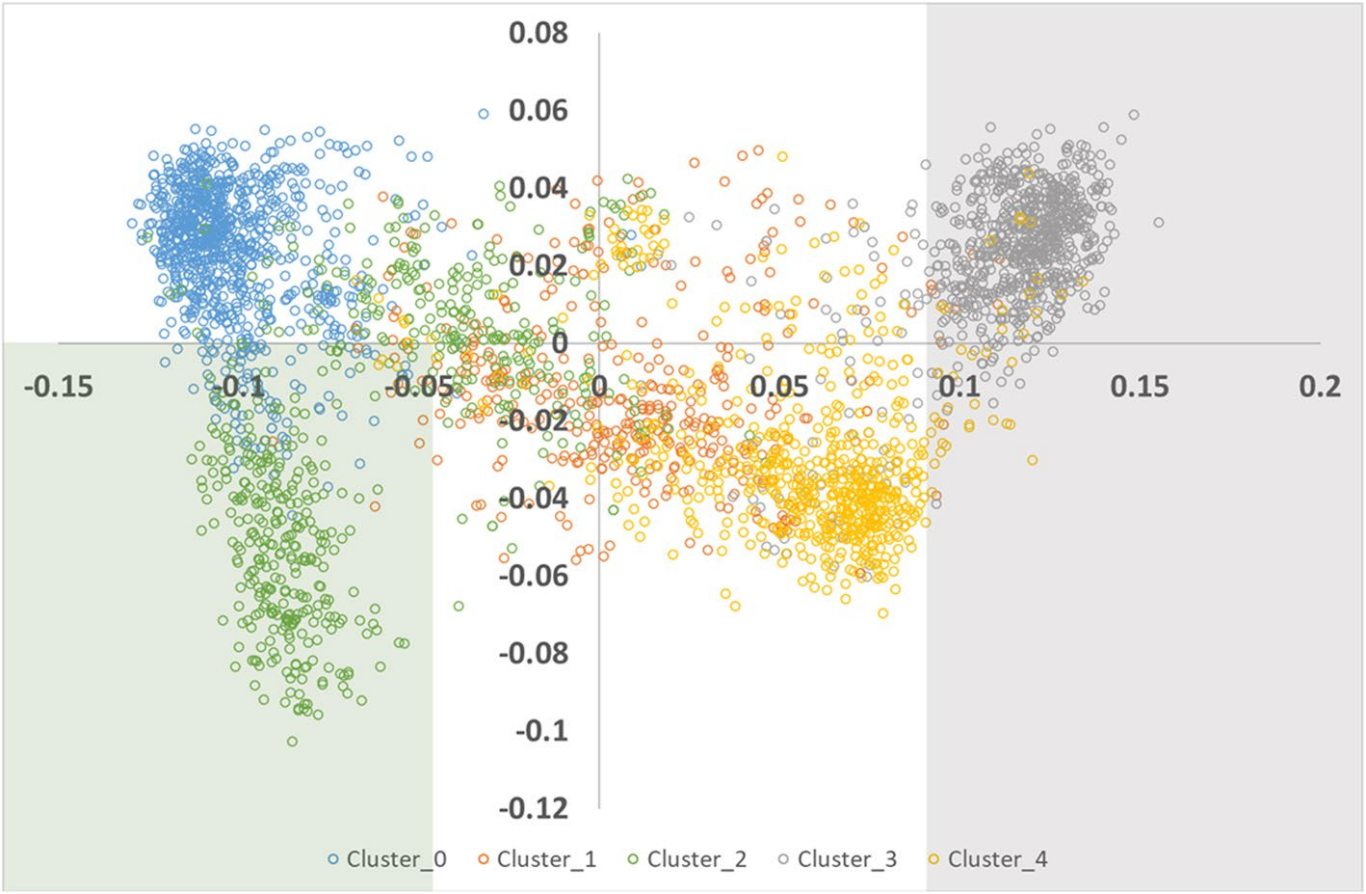
Multi-dimensional scaling of de-identified study participants (*N* = 3,226) colored by cluster with shaded regions identifying zones of refinement for cluster 2 (2A shaded in green) and cluster 3 (3A shaded in gray)

The multi-dimensional scaling (MDS) plot is shown in Fig. 3 and Figure S1. Genotype-based clustering in PLINK using the maximum cluster size option identified five clusters Fig. 3, Table S2.

The dispersion of 48 known locale-purpose individuals among the genotype-based clusters is described in Table 2. Certain dogs with known origins fell discretely into two clusters that clearly separated European exhibition (Cluster 0, N_identified_ = 11, N_unidentified_ = 886) and American non-exhibition/pet populations (Cluster 4, N_identified_ = 9, N_unidentified_ = 625). Cluster 1 identified animals that were likely hybridized between the European and American exhibition-bred groups (N_identified_ = 5, N_unidentified_ = 386) and could not be formally identified using our known purpose-locale animals (all known individuals informally bred from unknown or mixed locale). Cluster 2 (N_identified_ = 13, N_unidentified_ = 561) comprised both informally-bred and European working-line dogs. Cluster 3 (N_identified_ = 10, N_unidentified_ = 768) clearly comprised animals bred for exhibition. Most of the known-locale animals in Cluster 3 were located in the Americas with a further two located in Australia (Table 2).

**Table 2:**
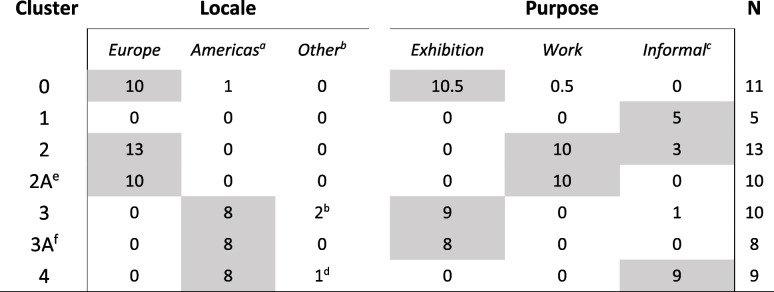
Dispersion of known locale-purpose individuals among DNA-based clusters identified by applying unsupervised DNA genotype clustering to 3226 unknown and 48 known locale-purpose dogs. Shaded cells represent the accepted region and purpose for each cluster

^a^Includes USA, Canada, Argentina

^b^Australia

^c^Includes pet, rescue, unknown, mixed

^d^Unknown locale

^e^Cluster 2 with multi-dimensional scaling (MDS) C1<-0.05, C2<0 for later analysis

^f^Cluster 3 with MDS C1>0.09 for later analysis

As a result of combining information from the MDS analysis with the newly defined clusters, we could readily observe that C1 from the MDS broadly describes the transition from European to American locale, while the C2 axis in the MDS broadly describes the transition from dogs bred for exhibition to those bred for other purposes including work or pet.

For DNA comparative analyses, Cluster 2 was refined to include only animals with MDS C1 < -0.05, C2 < 0 (N_identified_ = 10, N_unidentified_ = 309) to restrict the new cluster (Cluster 2A) to animals co-located with known European working dogs in the multi-dimensional scaling analysis Fig. 3.

Likewise, the initial Cluster 3 clearly comprised dogs bred for exhibition with most from the Americas. We further refined this cluster to exclude dogs known to be bred elsewhere (e.g., Australia) by restricting this group to those with MDS C1 > 0.09 (N_identified_ = 7, N_unidentified_ = 687) where the dogs based in the Americas were placed in the analysis. In later analyses we labelled this group for comparative purposes as Cluster 3A.

### Effective population size (Ne)

According to Waples et al. (2016) effective population size (Ne) determines the rate of evolutionary change due to genetic drift and informs the equilibrium level of genetic variation and the effectiveness of selection. The approximation of Ne provides population managers with a metric that can enable the population to be monitored to manage the loss of diversity and rate of inbreeding.

Initial filtering of the genotyping data for the 3,226 Dobermans resulted in the retention of 103,286 autosomal array marker loci. Genotyping success of less than 99% (–geno 0.01) removed 17,548 variants, while a further 87,007 variants were removed for low minor allele frequency (–maf 0.05). Local LD pruning resulted in the removal of a further 101,088 variants, resulting in 2,198 variants considered as unlinked in the primary data resource (*r*^*2*^ < 0.2). Later filtering to retain only variants polymorphic across all sub-cohorts resulted in the ultimate retention of 1,673 variants for analysis of *Ne*. During analysis, further filtering of resulting marker-pair LD values removed results where markers co-occurred on the same autosome, or where the observed r^2^ was higher than 0.2 (linked). This final filtering was unique to each locale-purpose cohort.

The estimated *Ne* from analysis of unlinked marker pairs for the entire sample cohort was *Ne* = 44. Values for *Ne* in locale-purpose cohorts are reported in Table 3. The refined formal locale-purpose cohorts (including European exhibition, Cluster 0; European work, Cluster 2A; Americas exhibition, Cluster 3A) defined by the multi-dimensional scaling analysis all demonstrated higher *Ne* relative to the entire data set providing strong evidence that the entire global cohort *Ne* value is under-estimated using the method applied.

**Table 3:** Effective population sizes for locale-purpose cohorts based on sample size adjusted inter-chromosomal linkage-disequilibrium

Locale-purpose	DNA Cluster	Cluster Size	Mean ^a^ r^2^	*Ne*
All	Global	3,226	0.0079	44
Europe exhibition	0	886	0.0030	177
Informal	1	386	0.0063	89
Europe working	2	561	0.0065	70
Europe working (refined)^b^	2A	309	0.0078	72
Americas exhibition	3	768	0.0034	158
Americas exhibition (refined)^c^	3A	687	0.0036	155
Americas informal	4	625	0.0030	236

^a^Adjusted for cohort sample size [8]

^b^Retaining individuals with C1 < -0.05, C2 < 0 from multidimensional scaling after unsupervised clustering

^c^Retaining individuals with C1 > 0.09 from multidimensional scaling after unsupervised clustering to exclude samples originating in Oceania

### Inbreeding and runs of homozygosity

The homozygosity analysis identified 551,586 individual runs of homozygosity (ROH). For any random region of the autosomal genome, the average percentage of dogs exhibiting a ROH including any random marker was 35.6% (range 0–98.5%, standard deviation 14.17%).

**Fig. 4:**
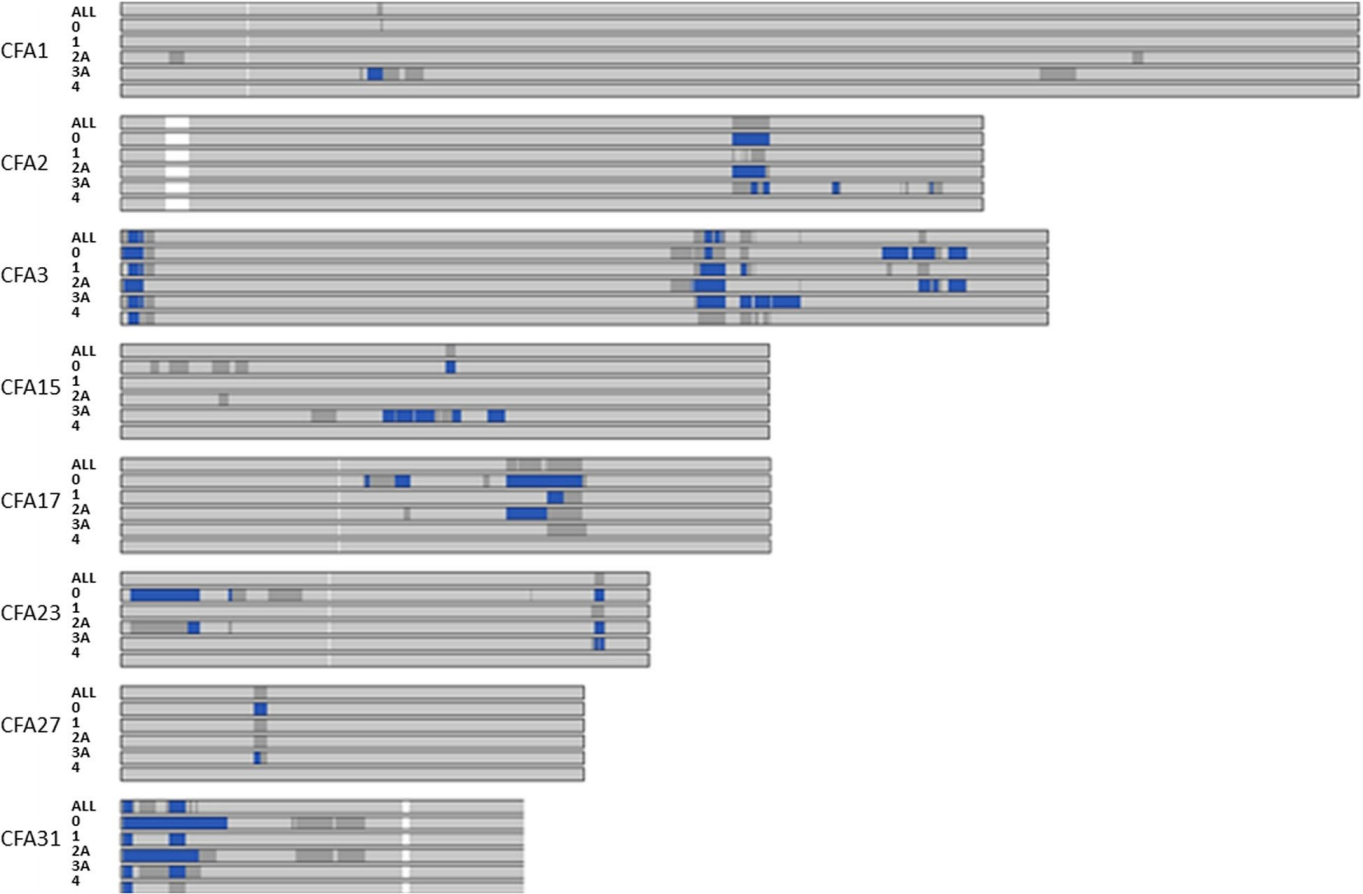
Eight Doberman chromosomes that exhibit regions with < 80% (light gray), >  = 80% homozygosity (medium gray) or >  = 90% homozygosity (dark blue) by locale-purpose cohort. Manual observation of the canine assembly suggests that uncoloured areas are likely reference assembly anomalies

Figure 4 depicts regions on eight chromosomes where either more than 80%, or more than 90% of the 3,226 Doberman samples had a concurrent ROH. Locale-purpose ROH regions for four sub-populations (Americas exhibition: cluster 3A, Americas informal: cluster 4, European exhibition: cluster 0, and European working: cluster 2A) are shown on the same image. Across the entire global Doberman population including the pet population, four genomic regions of larger than 1 Mb were fixed in 90% or more of the 3,226 dogs, comprising 5.2 Mb (0.23% of 2.2 Gb autosomal genome). The genomic regions were CFA3:0.8–2.3 Mb (1.55 Mb); CFA3: 57.9–58.7 Mb (0.8 Mb); CFA31:0–1.2 Mb (1.2 Mb); and CFA31:4.80–6.47 Mb (1.67 Mb). A total of 31 Mb (1.4% of genome) were included in ROH for more than 80% of individuals in the population including 11 loci on eight chromosomes.

Markers where at least one locale-purpose cluster showed more than 90% of members in a concurrent ROH are provided in Table S3. Of 7,517 array markers in such regions, 681 markers affected all locale-purpose clusters (Fig. 4, Tables S3 and S4). Later analyses considering fine-scale gene annotation focused on regions of >  = 500 Kb.

To test whether exhibition-bred animals had additional fixation relating to selection for breed standards, we focused on the merged exhibition group (cluster 0,3A). This analysis identified three further regions of strong fixation longer than 500 Kb: CFA2:60.6–64.3 Mb; CFA23:47.0 Mb-48.0 Mb; and CFA27:13.3 Mb-14.3 Mb. Of 7,047 array marker loci included in ROH for more than 90% of either European exhibition-bred (cluster 0) or Americas exhibition-bred groups (cluster 3A), only 777 markers were strongly homozygous in both group clusters (Table S4).

To test whether migration-based bottlenecks might influence fixation, we next focused on European animals derived from merged European exhibition (cluster 0) and the refined European working group (cluster 2A). This analysis identified no additional fixed regions but shared new regions identified in the exhibition-bred group, other than the CFA27 locus (Table 4). Of the 7,517 markers in regions with more than 90% of individuals from any locale-purpose cohort in an ROH, 5,734 markers were in ROH for more than 90% of either European working or European exhibition-bred groups. Of these, 2,340 array markers were in ROH for more than 90% of both the European exhibition-bred and European working bred Dobermans (Table S4). The number of markers with ROH for more than 90% of the merged European (exhibition and working) cohort (4,045 markers) was considerably greater than the equivalent number for the merged Americas and European exhibition cohort (1,844 markers). Thus, there is lower diversity within the European locale than within the exhibition purpose (Table S4).

**Table 4:** Regions with more than 90% of Dobermans in ROH^a^ by locale-purpose cohort

Chromosome	ROH start	ROH end	Array markers (N)	ROH Size (Mb)	Locale-purpose cluster(s)^b^
2	60,704,384	64,336,994	168	3.63	03A, 02A
3	778,939	2,325,941	126	1.55	Global
3	57,930,622	58,718,298	68	0.79	Global
15	32,931,140	33,274,592	33	0.34	03A, 02A
17	44,229,684	44,259,337	3	0.03	03A, 02A
23	46,996,102	48,025,418	90	1.03	03A,02A
27	13,280,578	13,840,836	46	0.56	03A
31	9,567	1,211,040	105	1.20	Global
31	4,803,675	6,469,309	138	1.67	Global
Total			777	10.80	

^a^ROH: Run Of Homozygosity

^b^Clusters are defined in Table 3

The strongest regions of differentiation between European (high-fixation) and American dogs (lower-fixation) affected CFA4:16.3-17 Mb, CFA11:63–64.8 Mb, and CFA16:40.5–41.2 Mb. Fine-scale variation in these regions was not assessed as the divergence was intermittent in the regions.

### High-resolution variant function analysis

Merging of the public variant call files yielded 1,257 unique dog identifiers and 8 unique Dobermans.

Regional fine-scale analysis follows. Where smaller genomic ROH regions for the global cohort are expanded within locale-purpose cohorts, results for the wider region are reported. The number of possible functional variants identified was 2,727 after multiple entries for the same locus were removed. Restricting attention to functional variants with Doberman FST of 0.7 or greater resulted in only 30 variants remaining. Further details relating to 2,727 variant sites are listed in Table S5. Attribute details for all observed variants in the regions based on 1,527 dogs represented in the public variant call files from whole genome sequencing are provided in Table 5. Attributes for the same variant set in eight unique Dobermans are reported separately in the same table.

**Table 5:** Attributes of variants in regions larger than 500 Kb observed as homozygous in 90% of Dobermans in any locale-purpose cohort. Variants are not filtered for FST

Chromosome	2	3	3	23	27	31	31
Region (Mb range)	60–64	1–2	57–59	46–48	13.3–13.8	1–1.2	4–6
Regional Variants ^a^ (1,527 dogs)	167,349	53,239	70,777	51,350	23,993	57,976	68,953
Polymorphic Variants (1,527 dogs)	166,008	52,629	70,303	51,035	23,871	57,492	68,445
Array ^b^ Markers (1,527 dogs)	4,375	812	4,765	3,615	315	4,230	934
Observed Functional Variants ^c^ (1,527 dogs)	713	429	854	312	57	298	64
Polymorphic Variants (8 Dobermans)	4,375	812	4,765	3,615	315	4,230	934
Polymorphic Array ^b^ markers (8 Dobermans)	32	5	76	48	1	3	-
Functional Polymorphic Variants ^c^ (8 Dobermans)	65	10	84	43	2	9	1
Monomorphic, Alternate allele ^d^, Functional ^c^ (8 Dobermans)	9	1	9	1	1	-	5
Locale-purpose clusters that have regions of concern	0, 2A, 3A	All	All	0, 2A, 3A	2A	All	All

^a^Markers are polymorphic in any one of 1,257 dogs

^b^Variants represented in array genotype data and variant call files from dogs with whole genome sequence

^c^Variants identified as putatively functional in the Jagannathan et al. variant call file (Jagannathan et al*.* 2019)

^d^Alternate allele is relative to the wider population consensus main allele and not the reference

#### CFA2

A large region on CFA2, CFA2: 60.7–64.3 Mb (~ 3.63 Mb) affected 90% ROH for clusters 0 (exhibition-bred Europe), 3A (exhibition-bred Americas) and 2A (working Dobermans from Europe). This genomic region accounted for 21 of the high FST variant alleles. Of these, 18 were missense variants, while a further three were variants in either 5-prime or 3-prime untranslated regions (UTR). All missense variants affected the gene *Carboxylesterase 1* (*CES1*) and were predicted to have moderate impact. Among the sequenced Dobermans, all 21 variant sites were polymorphic (Doberman DO263 heterozygous for 21/21 loci and DO159 heterozygous for 20 of 21 loci). All high FST *CES1* variant sites were very rare in the broader sequenced dog population.

Three variant sites affected the untranslated regions of genes *Iroquois Homeobox 6* (*IRX6*), *Iroquois Homeobox 3* (*IRX3*), and *Chromodomain Helicase DNA Binding Protein 9* (*CHD9*). The variants were predicted to be modifiers of gene action. At these sites all sequenced Dobermans were homozygous for the alternate allele.

Variant site coverage for Dobermans across the CFA2 region did not show any regions with differences in coverage that were suggestive of duplication or deletion relative to the broader sampled canine population (Table S6).

#### CFA3

Two regions were assessed on CFA3, CFA3:0.8–2.3 Mb (~ 1.55 Mb) and CFA3: 57.9–58.7 Mb (~ 0.8 Mb) affected the global Doberman population. The first region is close to the centromere. Seven high FST variant sites all occurred within the second region (CFA3: 57.9–58.7 Mb). Three missense variants were predicted to have moderate gene impacts on the genes *Mannosidase Alpha Class 2B Member 2* (*MAN2B2*) (2 variants) and *TBC1 Domain Family Member 14* (*TBC1D14*) (1 variant). The remaining variants affected the UTRs of *Ras Protein Specific Guanine Nucleotide Releasing Factor 1* (*RASGRF1*) (2 variants), *Cathepsin H* (*CTSH*), and *Protein Phosphatase 2 Regulatory Subunit B gamma* (*PPP2R2C*), each with a single variant. All UTR variants were predicted to be gene modifiers.

Marker coverage from the CFA3 regions showed no significant deviation between Doberman and other breeds (Figure S2, Table S6), other than one small region of potential deletion CFA3:1,204,000–1,205,000 that lies within a repeat element cluster that is intronic in the gene *Calcium/Calmodulin Dependent Protein Kinase IV* (*CAMK4*).

#### CFA23

The region taken forward on this chromosome (CFA23:46,996,102–48,025,418) affected all formally bred cohorts (0, 2A, 3A). Two putatively functional variants demonstrated FST values of higher than 0.7. These were a 3’-UTR variant in *LOC102151520* (polymorphic in Doberman) and a 3’-UTR variant in the gene *Ras-Related Protein Rap-2b* (*RAP2B*). Both variants are predicted to be modifiers. Doberman regional coverage was unremarkable.

#### CFA27

The region (CFA27: 13,280,578–13,840,836) was uniquely enriched for the exhibition-bred Dobermans (cohorts 0 and 3A) contained no putative functional mutations with strong FST (> 0.7). Low diversity in the CFA27 region may indicate differential selection for desirable traits or may result from genetic drift. Three potential functional variants were identified in the gene *Leucine-Rich Repeat Serine/Threonine-Protein Kinase 2* (*LRRK2*). One monomorphic alternate variant was a SNP in the 5’-UTR. Two further Doberman polymorphic variants affected a splice-region (FST = 0.3) and the 3’-untranslated region (FST = 0.38) of *LRRK2* (Table S5). Sequence alignment coverage over the region was not unusual.

#### CFA31

Marker coverage data from the CFA31;1–1.2 Mb region in the variant call file for eight sequenced Dobermans suggests that this part of the genome may be subject to a significant structural variant with copy number expansion of perhaps up to 3.6-fold (mean regional coverage of 56 reads at CFA31:30,000–69,000 compared with a mean cover of ~ 18 reads elsewhere (Figure S2, Table S6). The importance of the apparent copy number expansion was not determined or further explored in this analysis. Since the region is close to the centromere the higher level of cover may signify centromeric satellite sequence expansion. The Doberman excess read coverage in the region was unremarkable in relation to the wider sequenced dog population. The broader CFA31 ROH region includes the potential behaviour gene *5-hydroxytryptamine receptor 1F* (*HTR1F*) (serotonin receptor) at CFA31:272,358–274,597 (canFam 3.1) where Dobermans exhibit the reference allele at an annotated variant site.

## Discussion

Purebred dog populations and, thus, their population management are influenced by the registry in each region. In certain countries, registries allow for controlled importation and breeding of foreign animals. The American Kennel Club allows such importation, breeding, and registration of subsequent puppies depending on a foreign dog’s country of origin. This has allowed for relatively free flow of foreign dogs into the Americas. However, other registries, such as Germany’s Dobermann-Verein, have in past times adopted policies that effectively disallow breeding of imported dogs and prevent recognition of resulting puppies [9]. Additionally, the American Kennel Club designates all dogs descended from an albino female Doberman with a unique “WZ” registration, regardless of the puppy’s coloring or genetic status. This designation allows for easy identification of possible albino ancestry and presumably discourages breeding [10]. Thus, interbreeding among the Americas-exhibition, European-exhibition, and some European-working, and Americas-pet cohorts is technically allowed but not commonly performed for various reasons. Despite this, there is no functional barrier preventing the interbreeding of any of these groups and resulting puppies may be registrable depending on country and individual registry regulations, which are subject to change.

### Population dynamics

The higher *Ne* within cohorts is a result of lower mean r^2^ in the within-cohort groups and was not driven by the correction applied for sample size. This phenomenon is recognised as a signature of population admixture and suggests that the global cohorts of Doberman pinschers can be regarded as genetically distinct populations for analytical purposes [11]. The sub-cohort effective population sizes are therefore expected to better reflect the true characteristics of those individual populations.

It is rare that such a large single breed cohort is available for population analysis. The outcomes of this analysis challenge our perceptions of accepted methods for determining *Ne* using genotypes, and in particular the common belief that DNA-based assessment is always superior in accuracy to pedigree-based estimates. The reduced estimated *Ne* in the global cohort relative to the individual sub-groups using the analysis of unlinked inter-chromosomal markers shows that the estimation of *Ne* using admixed population samples can generate significant analytical biases that manifest as an underestimation of *Ne* in the admixed group. The within-cohort *Ne* values are likely closer to the true values. A previous pedigree analysis of UK exhibition-bred Doberman pinschers revealed an *Ne* of 133.4 but with declining rate of inbreeding since the year 2000 that is suggestive of increasing *Ne* [12]. The observed UK value supports the values reported for the exhibition-bred cohorts in the current study that possibly originate from the Americas gene pool. Unlike pedigree-based population genetics analysis, DNA-based analyses represent a snapshot in history at the time of sample collection. The lower *Ne* observed in the European working line dogs may well relate to the historical population bottlenecks occurring around the time of World-War II as reported by the Veterinary Genetics Laboratory (VGL), University of California, Davis report [13]. Using microsatellite analysis, the VGL report showed very low values for *Ne*. However, because the values for *Ne* were calculated separately for each locus, it is uncertain how the VGL reported *Ne* statistics relate to measures based on LD between unlinked loci. Another possible reason for lower *Ne* in the European cohort may relate to region of origin biases in breeding programs. It is possible that because the breed originated in Europe, European breeders may be reluctant to include genetics from foreign-bred animals thus reducing the capacity for maintaining diversity. Different breeding norms between Europe and the Americas may also contribute to this, since it is likely that European breeders will include consideration of tail and ear phenotypes that are less important in countries where dogs are routinely docked.

### Genomic regions diverged by locale

Geographic locale was observed to exert a greater influence on genomic fixation than breed purpose in the Doberman (Table S3 and S4). Regions on CFA4, CFA11 and CFA16 were particularly diverged by geographic locale.

The CFA4 region that has low fixation in the Americas and high fixation in the European animals includes potential long non-coding RNAs and is near the gene *catenin alpha 3* (*CTNNA3*), while the region on CFA11 that is similarly diverged between European and American-bred dogs also contains a gene from the catenin family: *catenin alpha-like 1* (*CTNNAL1*). A related gene *catenin alpha 2* (*CTNNA2*) was previously identified as a potential candidate for obsessive behaviour in the breed [14]. The CFA16 region contains genes that may influence cardiac and neoplastic disorder risk including *microtubule-associated tumor suppressor 1* (*MTUS1*) and *fibrinogen-like protein 1* (*FGL1*). Fine-scale variation was not assessed in the diverged regions.

### External phenotype and locale-purpose

Using array markers, rather than whole genome sequencing data, it was not possible to directly ascertain the locale-purpose prevalences for the mutations with genetic tests that are offered for the breed. However, an approximation of the prevalence of off-standard white/cream coat color related to genotype at the gene *solute carrier family 45 member 2* (*SLC45A2*) could be made through observation of allelic calling of two markers contained within the deletion mutation (CFA4:73,867,685C > Del and CFA4:73,870,657G > Del) and heterozygosity could be assessed through observation of two further loci in strong LD (CFA4: 73817338A > G and CFA4:73912726G > A). Of 44 animals observed to have two copies of the deletion haplotype, 30 occurred in Cluster 4 (Americas informal/Pet), one in Cluster 3A (Americas exhibition) and the remainder had initially been included with the Americas exhibition cluster (Cluster 3) but were excluded by refinement based on the multi-dimensional scaling.

Short-tailed phenotype (Natural bobtail) dogs [OMIA 000975–9615] could be desirable among exhibition competitors in countries that do not allow tail-docking. One mutation in the *T*-gene is known to code for the phenotype as a semi-dominant gene that is lethal as a homozygote [15, 16]. It has been suggested that there may be further undescribed mutations affecting the existence of the phenotype [16]. We observed low frequency heterozygous genotype calls and no alternate homozygous calls for one marker in the vicinity of the *T*-gene (marker BICF2S23215117 at CFA1: 54,199,967). The concordance of this genotype with any tail phenotype could not be directly assessed but the heterozygous calls occurred predominantly in the informally bred clusters (Cluster 1: 25 heterozygotes, and Cluster 4: 15 heterozygotes). Four heterozygous dogs fell into Cluster 0 (European exhibition) and three in Cluster 3A (Americas exhibition—refined).

### Disease genomics

The Doberman has been identified as a breed that has an elevated risk of premature death related to cardiac disease (particularly dilated cardiomyopathy or DCM) [OMIA 000162–9615], cancer (particularly lymphoma [OMIA 001733–9615] or osteosarcoma [OMIA 001441–9615]), and CS (wobbler syndrome) [OMIA 001894–9615] [17–19]. The analysis of the public phenotype data accessed by the current study supports that the named conditions are truly important reasons for mortality in the breed.

*Cardiac disease – congestive heart failure (CHF) and dilated cardiomyopathy (DCM):* Cardiac disease is by far the most significant cause of premature death in the breed, accounting for an average of 28% of deaths in the sampled cohort (Table 1). In the sampled phenotypic data, significantly elevated mortality from cardiac disease was demonstrated by European dogs bred for both work and exhibition relative to the global mean. From the phenotypic analysis, the European dogs had a two-fold higher risk of death from cardiac disease than dogs bred in either Australia or South Africa, although samples from the latter two countries were limited and so this finding should be interpreted with caution.

Commercial genetic testing is currently offered for DCM in Dobermans at two loci. The first tested locus considers a mutation that is a 16-base pair deletion in the 5' donor splice site of intron 10 of the *pyruvate dehydrogenase kinase 4* (*PDK4*) gene (NM_002612.4 chr14:20,827,965–20,838,970). Functional evidence [20, 21] suggests that the mutation affects the response to mitochondrial stress in affected cells. In-vitro, cells that are heterozygous or homozygous for the *PKD4* deletion demonstrate evidence of mitochondrial stress and activation of the intrinsic apoptotic pathway following cell line starvation [21]. To date, further studies have failed to validate the *PKD4* association in the breed [22]. A second locus was identified by the same research group in an affected family of Dobermans that was clear for the *PKD4* mutation. This family segregated a coding mutation in the gene titin (*TTN*, NM_001329611 chr36:22,146,870–22,417,858) which is a major candidate gene for the condition based on human studies [20]. The percentage of dogs with ROH at the *PDK4* locus was 24% and was 23–31% at the *TTN* locus (Table S3). Both loci have lower than average numbers of dogs exhibiting ROH. Therefore, *fixation* at these loci is unlikely to be the reason for high levels of DCM in the breed. However, the *PDK4* and *TTN* loci may segregate to add to DCM risk in individuals and families. The results of the current analysis provide no information as to the importance of the two loci in the population levels of DCM.

The current analysis has identified promising targets for further research into heart disease in the breed. Among the locations identified as having low diversity and high FST in the breed, the low diversity region on CFA2 contains multiple genes with potential cardiovascular function. According to the Genecards resource [23] *CES1* has a demonstrated role in cardiac conduction and cardiac drug metabolism while *IRX6* is a potential hub gene in the pathogenesis of heart failure [24]. *IRX3* is potential modulator of cardiac copper metabolism [25]. On CFA3, the gene *MAN2B2* in Doberman populations contains two potentially damaging missense variants that are very rare in the general dog population. *MAN2B2* has been reported as a potential early biomarker for cardiac hypertrophy and heart failure in an isoproterenol-induced model of cardiac hypertrophy in the mouse [26].

*Cervical spondylomyelopathy (CS):* It has been reported that neonatal Doberman pups are born with abnormal structure of their cervical vertebrae and that the breed is over-represented among those affected by CS [18]. However, to date no study has shown a conclusive heritability for CS risk in the breed [17]. In the phenotypic data analyzed here, CS was responsible for approximately 6% of deaths reported. The observation of breed elevated risk of CS without obvious within-population heritability may suggest the enrichment of predisposing genetic factors in the breed by selection or genetic drift. It is possible, although not discussed in the literature, that the upright stature and head carriage of the breed may be associated with underlying genes that predispose to the condition. However, if the desirable breed external phenotype increased risk for CS, we would expect to observe an increased prevalence of Wobbler syndrome among animals bred for exhibition. That trend was not observed in the phenotypic data. Of the major locale-purpose cohorts represented in the phenotypic data, the risk of CS was highest in Australian Dobermans with 10.5% of mortality attributed to CS (perhaps influenced by sampling error from poor group representation in the survey) and in USA informally bred Dobermans with 10.3% of mortality due to CS. To date, there is no commercial genetic test that predicts CS risk in the breed. European bred dogs, regardless of breed purpose, exhibit lower than expected CS-mortality. No obvious predisposing candidate genes for CS were identified in the strong ROH regions, however, the region on CFA4 that is diverged between American and European dogs contains a retrocopy of the gene *Single Stranded DNA Binding Protein 3* (*SSBP3*). According to the University of Santa Cruz genome browser, the primary *SSBP3* gene on CFA5 may play a role in the development of spinal interneurons. If retaining any function, the retrogene may have potential to influence geographically linked risk of CS. If the retrotransposed gene has relevance to this disorder, then it is more likely that a deleterious haplotype in the region has been selected against in the European dogs.

*Neoplasm (Osteosarcoma, Lymphoma, and Hemangiosarcoma): *In the phenotypic data, the different locale-purpose cohorts appeared to segregate different predispositions to cancer (Table 1) which collectively accounted for around 14% of deaths. European dogs exhibited lymphoma more frequently than the Americas derived dogs, whilst hemangiosarcoma was unreported in Europe and highest among exhibition-bred dogs in the Americas. The occurrence of osteosarcoma was lowest in the European working bred dogs. Recent evidence suggests that hemangiosarcoma risk may be linked to persistent infection with the bacterium *Bartonella spp* that is transmitted via blood sucking parasites including ticks and fleas [27]. The relative prevalence of canine infection with *Bartonella spp* in Europe versus the Americas is not known so it is not possible to determine whether a genomic or local environmental difference may underlie the differential risk for hemangiosarcoma.

On CFA16, the geographically diverged region contains multiple genes that may influence geographically diverged disorder risk. According to the mouse phenome browser [28], the gene *MTUS1* is linked to both cardiac hypertrophy and B-cell lymphoproliferative disease in the mouse [29]. In the same region of geographic divergence,* FGL1* is a major ligand of *Lymphocyte-activation gene 3* (*LAG3*) that inhibits antigen-specific T cell activation. In mice, knock out of *FGL1* promotes T cell immunity [30] and this might have relevance to T-cell based lymphoma.

### Lifespan

The phenotypic data used in this analysis suggest that the life expectancy of a Doberman at birth is 9.14 years. An earlier analysis of UK companion dog data showed that the mean life expectancy for companion dogs (across breeds and mixes) under primary veterinary care in the UK was 11.2 years [5]. Eighteen breeds were included in the Teng analysis excluding Dobermans. The 2014 British study [7] reported a lifespan of 7.67 years for the Doberman breed, however, that result was obtained from far fewer dogs (90 participants) and so may have been subject to sampling error.

Growing evidence suggests that organismal lifespan is shortened by deficits in autophagy [31, 32]. The second ROH region on CFA3 (CFA3: 57.9–58.7 Mb) includes a missense variant in the gene *TBC1D14*. *TBC1D14* controls regulation of autophagosome assembly, the recycling of endosome to Golgi transport; and is a negative regulator of autophagy. Several further variants affecting the gene and this region did not meet the FST cut-off but may contribute to complex phenotype interactions arising from this locus.

The phenotypic data suggest that inbreeding depression almost certainly plays a role in reducing lifespan in this breed. The European born animals, regardless of breeding purpose, exhibited lower life expectancy than other locale-purpose cohorts. The European working bred Dobermans have the shortest life expectancy and the lowest effective population size of all groups. Based on observations of animals in the phenotypic analysis, crossing European working line dogs with the dogs from elsewhere (European exhibition bred or Americas bred dogs) is expected to improve life expectancy (Fig. 1). There may be reluctance to use outcrossing if it is considered that out-breeding will diminish the working potential of the resulting pups. This dilemma is common in breeds that have breed splits based on breed purpose. If the major impacts on lifespan are being generated by the genes and genomic loci observed as regions of concern in this study, then it may be possible to use targeted crossing programs to maintain the desirable working characteristics while alleviating the major negative influences of the identified loci. Similar programs have been applied in other breeds (e.g., Dalmatians to alleviate hyperuricosuria risk [33]. Gene targeted crossing programs that apply out-crossing followed by back-crossing have potential to maintain the essential or highly desirable characteristics of the animals while improving welfare outcomes. This might be possible without using other breed contributions but is likely to be more difficult.

It is also possible that work-purpose bred dogs may have reduced life expectancy due to wastage (election for euthanasia at the end of work life). There was little evidence to support this as a potential cause in the phenotypic data. The major causes of death in the group were: cardiac disease 36%, cancer (various) 32% and other (13%). Figure 2 shows that the “other” category for death appears to be reasonably constant through dog ages.

### General discussion

The analysis of gene function applied here relies on previous functional annotation provided by the Dog Biomedical Variant Database Consortium [34] and may not be exhaustive. However, already we have identified target genes and variants that might play a role in the expression of complex disorders that have high prevalence in the breed and impact observed longevity.

Typically, genotyping array markers are polymorphic in many populations of animals, meaning that the included variants arose prior to breed development. Variants arising at later times may not be visible using the older variants as markers. For this reason, although we have identified four regions with strong ROH across the global Doberman population, fine-scale genomic analysis shows that variation (including potential functional variation) exists in the polymorphic state within the breed within most of these regions. Although marker-based evaluation in such regions is unlikely to be effective unless it is based on the direct genotyping of functional mutant sites, phenotype-based selection can still act on hidden allelic frequencies of younger variants.

Where potential deleterious variants are identified in low-diversity regions, it is critical to identify which haplotype harbors the deleterious allele. It is possible that where phenotypic selection has reduced diversity in the region to remove a deleterious gene variant, the rarer haplotype might be damaging to health. Population managers must ensure that the frequencies of deleterious variants are not increased in attempts to improve statistical diversity for a given region.

In many cases in this analysis, the fixed or most common allele was the global main variant (likely the ancestral normal variant). An example where attempts to improve diversity may inadvertently create problems is the gene *MAN2B2*. At this locus we identified two missense variants that have FST > 0.7. The ancestral haplotype is CFA3:58673093C, CFA3:58696797 T (CT). Observed Doberman haplotypes were: CFA3:58673093C, CFA3:58696797 T (CT); CFA3:58673093C, CFA3:58696797 T > C (CC); and CFA3:58673093C > G, CFA3:58696797 T (GT). In Dobermans the most common haplotype observed was CC (one missense variant), the second most common haplotype was GT (two-missense variants). Thus, selecting to increase the frequency of the lower frequency haplotype in this instance may inadvertently increase the deleterious mutation load on the gene. At this gene locus, the wild-type haplotype was present only as a single heterozygote in the sequenced Dobermans. Table S5 provides information on many putative functional variant sites across the ROH regions of concern. Careful navigation will be required to understand the individual and combined impacts of the variants on gene function and clinical outcomes in the breed. That depth of analysis was not attempted in this study.

## Conclusions

The analysis here agrees with other published results that demonstrate high levels of homozygosity for genotyping array markers in the Doberman breed. Despite high general levels of homozygosity in the breed, surprisingly few regions of the genome are “fixed” (denoted as regions of concern). Less than 1% of the autosomal genome is depauperate of variation as defined by more than 90% of individuals with a concurrent ROH and less than 2% of the genome (31 Mb) has fixation at the 80% ROH level.

Genetic divergence between the Americas-exhibition and European-exhibition locale-purpose groups means that hybridization between the North American and European exhibition-bred animals has potential to alleviate fixation and improve heterozygosity at many (though not all) problematic gene loci in regions of concern. This should be possible given current accepted breeding practices in the registered dog world. The same opportunity is less likely to generate substantive improvement for hybrids between the European working and European-exhibition groups as there is strong genetic concordance among the European locale-purpose groups in this analysis. Animals within the Americas pet/informal population may provide a further reservoir of diversity that can be used to alleviate diversity loss in genomic regions of concern.

Functional analysis of regions of concern has provided a rich resource for further research that may improve clinical outcomes in the breed. Genes within the regions have potential to impact important disease and life outcome processes such as cardiac health, cancer and lifespan as influenced by autophagy.

## Methods

### Geographic cohort-based phenotyping data

A citizen-science based breed survey (dobehealth.org accessed 7/9/2021) collected cause of death for 1,744 Doberman dogs between years 2010–2021. The data are freely available in the public domain and include dogs identified by participating owners as being from Europe, North America, Africa, and Australia. It is possible that some dogs are represented in both genotyping and phenotyping data but as the animals are de-identified it is not possible to effectively match individual participants.

All dogs with recorded age of death contributed to a life table constructed using the age at death data reported [5, 35]. Where animals had two concurrent disorders at the time of death, the primary cause of death was the disorder requested.

Causes of death contributing more than 50 deaths in the data resource—hereafter termed leading causes of death, were identified. The data were first cleaned to exclude individual reports with missing or impossible age of death and counts of leading causes of death at each whole year of age were identified. Animals less than one year old were attributed an age of zero. Animals older than 18 years were excluded. Deaths caused by “old-age”, “other” or “cancer-other” were excluded from further analysis.

Data relating to remaining leading causes of death were next analysed to determine the percentage of animals from each geographic region affected by any leading cause of death. The percentage occurrences of individual and grouped leading causes of death in each region were compared. Geographic regions exhibiting over- or under-representation for the cause of death were identified. Over- or under-representation was based on the mean geographic region percentage of animals affected by the individual or grouped leading cause and the 95% confidence interval (i.e. ± 1.96*s.e).

### Genotyping array data

The Doberman Diversity Project is a citizen science-based project devised by Sophie Liu and Robin Loreth in collaboration with Embark Veterinary. The project obtains data from Illumina Canine HD genotyping arrays that are gathered through routine genetic testing of collaborating dogs with the consent of the owners. The de-identified data are released for analysis. With owner consent, genotyping array data from a further 48 individuals with known geographic and breed-purpose origins were released to enable the attribution of geographic cluster to the wider genotyping array sample. These known samples were used only to assign geographic location and purpose (locale-purpose) to DNA-based clusters and were then excluded from the broader analyses.

### Locale-purpose DNA profiles

Locale-purpose attribution was determined by two methods, first, individual multi-dimensional scaling coordinate proximity to 48 animals with known geographic location and breed purpose (locale-purpose) was determined. To do this, the first and second principal components were derived from multi-dimensional scaling analysis in PLINK following standard quality filtering of autosomal array data: [minor allele frequency (maf) –maf 0.05; genotyping fail rate –geno 0.1]. Clustering (–cluster) and multi-dimensional scaling plots (–mds-plot 2) were generated for autosomal chromosomes (–chr 1–38). The resulting variance component coordinates (C1 and C2) were used to ascertain two-dimensional coordinate values. Animals from the de-identified set (*N* = 3,226) were assigned to groups based on common clustering with the animals of known-origin in an analysis that included the 48 known-origin samples and with the maximum cluster size set at 900 to force separation [ –geno 0.01 –cluster –mc 900].

### Effective population size

Assessment of effective population size (*Ne*) can be made using the observation of residual pairwise-linkage disequilibrium (LD) in otherwise unlinked SNP markers. We generated a set of unlinked markers that could be used to estimate *Ne* following previously described methods [8, 36, 37]. To achieve this, array variants for the entire sample group were manipulated in PLINK [38] to prune markers with LD of *r*^*2*^ > 0.2 in 100 kilobase windows with a single marker step-size (–indep-pairwise 100 1 0.2), and to include only autosomal markers (–chr 1–38). Minor allele frequency (maf) (–maf 0.05) and stringent genotyping rate (–geno 0.01) filtering were employed to further reduce the size of the marker set. The allele frequency estimation option in PLINK was used to ascertain the minor allele frequency of the “prune.in” variants that were deemed as unlinked in the first pass analysis for each individual cohort group.

Next, PLINK was used to assess the pair-wise LD (–r2 inter-chr) on a reduced set of markers retaining those from the “filename.prune.in” list with maf > 0.05 in every cohort. The allowable observable LD in the scanning window was reduced to zero (–ld-window-r2 0). Resulting pairwise r^2^ values from the presumptively unlinked marker pairs were averaged across the data but restricted to marker pairs located on different chromosomes and marker pairs with *r*^*2*^ < 0.2 to avoid artefacts caused by possible reference genome assembly errors. Only markers that were polymorphic in all locale-purpose groups were retained to prevent biased r^2^ estimates resulting from having one marker in the marker pair fixed.

The equation of Waples (2006) [36] was used to ascertain *Ne*:

$$Ne=\frac{1}{3({{r}_{adj}}^{2}-\frac{1}{S})}$$

where $Ne$ is the effective population size, S is the sample size (e.g. S = 3,226), and ${{r}_{adj}}^{2}$ is the sample-size adjusted mean ${r}^{2}$:

$${r}_{adj}^{2}={r}^{2}{[\frac{S}{\left(S-1\right)}]}^{2}$$

following the equation of Weir (1979) cited in [8]. In the equation, ${r}^{2}$ is the unadjusted mean inter-marker association, ${r}_{adj}^{2}$ is the adjusted mean ${r}^{2}$ and, S is the sample size (e.g. S = 3,226 for the global cohort).

The same files and parameters were re-analyzed for individual locale-purpose subsets of the data (European exhibition, European work, American exhibition, and American pet populations) to assess intra-population *Ne*.

### Inbreeding and runs of homozygosity (ROH)

The Doberman Illumina canine high density array genotypes were used to conduct genome-wide analysis of breed homozygosity and to obtain any evidence for selective sweep in the breed. The main analysis was conducted using PLINK employing the running parameters [–homozyg-snp 50 –homozyg-window-het 1 –homozyg-window-missing 1 –homozyg-gap 200 –chr 1–38]. These parameters define the number of markers in the scanning window frame (–homozyg-snp), the maximum allowable number of heterozygous markers in a segment to accept it as homozygous (–homozyg-window-het), the maximum allowable number of missing markers in a homozygous segment (–homozyg-window-missing), and the maximum allowable gap between adjacent markers in a homozygous segment in kilobases (–homozyg-gap 200. Diversity statistics are reported for autosomal markers only (–chr 1–38). The same analysis was repeated for the defined locale-purpose subsets of the data.

### Identification of regions of concern

Genomic regions exhibiting strong levels of homozygosity suggesting selective sweep or paucity of variation (*regions of concern*) were explored in detail. Analysed population cohorts included the global cohort, individual locale-purpose cohorts, and two merged cohorts. For each analysed cohort, the arbitrary cut-off for further analysis of an individual locus was having 90% of cohort individuals within a common run of homozygosity (ROH) and a region size of > 500 kilobases (Kb). The region size was defined by the inter-marker distance between the first and last markers in an ROH exhibiting at least 90% cohort individuals homozygous. All ROH coordinates are reported relative to canFam3.1 [39]. Genotype frequencies for each individual array marker (as opposed to ROH segments) were calculated using the –freq-x option in PLINK [38]. Note that ROH segments for individual animals can include occasional heterozygous or missing markers according to the running parameters.

In addition to the global merged group, two merged locale-purpose groupings (*co-managed clusters*) were considered. First, we focused on loci with more than 90% ROH in locale-purpose groups that commonly allow interbreeding (i.e., the merged locale-purpose cluster 03A that combines European animals bred for exhibition with exhibition animals from the Americas). This grouping was chosen to enable better management of individuals that can be freely interbred using standard international conventions. The second merged locale-purpose group considered was that of the European animals (comprising animals bred for exhibition and for work in Europe). This grouping enabled the potential evolutionary influences of geographic breed bottlenecks to be better examined.

### High-resolution variant function analysis in regions of concern

Regions of concern (defined as at least 90% of cohort dogs in a concurrent ROH) were explored in higher resolution using public variant call file (vcf) data resources [34, 40]. To do so, ROH region variant calls were first extracted from the vcf for 722 diverse dogs and wild canids from [40] using the TABIX tool [tabix -h 722 g.990.SNP.INDEL.chrAll.vcf.gz chrN:region_start-region_end > 722_region_vcf] [41]. The same procedure was used to extract regional data from 590 dogs and canids from [34] [tabix -h 590_220K_array.vcf.gz N:region_start-region_end > 590_region.vcf] where N is the chromosome number.

The regional vcf files from Jagannathan et al. (2019) and Plassais et al.(2017) were next merged using PLINK [38] and converted to ped and map format. The map file was recoded to show each marker identifier as a combination of the chromosome and base position. This avoided many markers having the same marker identifier (otherwise they were recorded as typically either “.” or their rs-code).

The vcf data from the [34] study is coded relative to the canFam3.1 reference assembly and includes functional prediction as a component of the vcf file information field for each variant called. The “INFO” field within the vcf was interrogated to identify variant positions that generated potentially functional variants using the command-line tool *grep* [grep (missense, codon, splice, frame, prime) 590_region.vcf > 590_region_functional.vcf]. From the resulting functional variant file, variant positions and functional annotations were extracted.

To test the hypothesis that strongly diverged Doberman breed alleles in the regions of low diversity are damaging, pairwise *FST* (Doberman versus non-Doberman) was calculated using PLINK (options –fst case–control). The *FST* compared whole-genome sequenced identified Doberman genotypes with those of all other dogs represented in the large merged vcf for each individual ROH region. Variants annotated as putatively functional that demonstrated high frequency derived alleles (pairwise *FST* >  = 0.7) were analyzed further. Potential error regions exist when all sequenced animals (including non-Dobermans) have the alternate variant when successfully genotyped. The likelihood of Doberman relevant phenotypic expression for individual identified genes was evaluated through literature review of the gene name in relation to the leading causes of death in the breed.

Regional sequence coverage was assessed for biallelic markers in the ROH regions with >  = 90% individuals in the ROH. Read coverages for the eight whole-genome sequenced Doberman individuals were determined by the allele depths derived from the individual genotype calls in the combined vcf. Allelic coverage values for the eight sequenced Doberman individuals were averaged to generate a breed mean sequence depth metric for the breed at each biallelic marker. The per-variant coverage was assessed in ten kilobase windows.

## Supplementary Information

**Additional file 1.**

## Data Availability

Electronic information relating to Doberman lifespan and cause of death are available from: https://dobehealth.org/results/index.php Access to genotyping data is provided on request from the Doberman Diversity Project: https://dobermandiversityproject.org/
